# A quadratic correlation between long‐term mean group size and group density in a cooperatively breeding passerine

**DOI:** 10.1002/ece3.3405

**Published:** 2017-09-18

**Authors:** Dian‐Hua Ke, Yan‐Hui Deng, Wei‐Bin Guo, Zu‐Hao Huang

**Affiliations:** ^1^ School of Life Sciences Jinggangshan University Ji'An China; ^2^ Library of Jinggangshan University Jinggangshan University Ji'An China

**Keywords:** cooperative breeding, *Garrulax perspicillatus*, habitat quality, masked laughingthrush, quadratic correlation, social structure

## Abstract

Both mean group size (MGS) and mean group density (MGD) are critical indices to characterize a population of cooperatively breeding birds. When a population reaches its carrying capacity, both long‐term MGS and long‐term MGD will remain relatively stable. However, there has been little study of how these two variables relate. The Masked laughingthrush *Garrulax perspicillatus* is a cooperatively breeding bird living in fragmented habitats. During 2010 and 2012‐2016, we used song playback to observe and confirm the group sizes and territory ranges of the birds and the data of bird presence to determine habitat suitability. By grouping the nearest territories according to their geographical coordinates, we divided the whole study area into 12 subareas and the whole population into 12 subpopulations. Then, we calculated both MGS and MGD for different time durations for each subpopulation. Finally, using MGD as independent variable and MGS as the dependent variable, we explored the correlations between MGS and MGD by fitting quadratic functions and modeling quadratic regression. Both MGS and MGD were averaged for different time durations and were cross‐related. Our results show that the MGS for more than 2 years significantly correlated with MGD for more than 3 years in a reverse parabolic shape, differing from that of short‐term effects. Our findings suggest that long‐term MGD is a better predictor of long‐term habitat quality and that long‐term MGS is determined by long‐term habitat quality in Masked Laughingthrushes. Based on above findings, we can infer that: (1) Long‐term habitat quality determines the long‐term MGS, but it sets no prerequisite for the status and source of group members; (2) Long‐term MGS in certain populations is adapted to the corresponding level of long‐term habitat quality, it facilitates us to predict the helper effects on current or future survival or reproduction in different situations. These findings and inferences are both helpful for us to understand the evolution of cooperative breeding.

## 1 INTRODUCTION

Cooperative breeding is a reproductive system in which more than two individuals show parent‐like behavior toward young of a single nest or brood. Although the social structures of these systems vary across species or populations, from helping by close relatives to cooperative polygamy or plural breeding, cooperatively breeding birds usually live in groups of two to 15 individuals (Stacey & Koenig, 1990; Ke, Griesser, & Huang, 2016), with mean group sizes of <10 individuals (Smith, 1990). The mean group size (MGS) can be used as an indicator of the prevalence of helpers in a cooperatively breeding population (Stacey & Koenig, 1990; Koenig & Dickinson, 2016). For certain cooperative populations, the larger group size suggests the higher popularity of helpers. In this case, it is crucial to explore how the group size varies with ecological factors.

The MGS of cooperative breeders was reported to be related to ecological harshness (prey abundance or rainfall; Emlen, 1982; Stacey & Koenig, 1990; Russell, 1999; Ke, 2009). For example, the MGS in Seychelles warblers *Acrocephalus sechellensis* was larger in high‐quality territories (Komdeur, 1992); groups of the Superb starling *Lamprotornis superbus* were larger in years with greater rainfall during the prebreeding period (Rubenstein, 2016). In addition, abundant insect prey or rainfall in the month preceding breeding led to a lower proportion of population helping in White‐fronted bee‐eaters *Merops bullockoides* (Emlen, 1982), and extremely low rainfall led to a higher proportion of helping in the next year in Tibetan ground tits *Parus humilies* (Ke, 2009). Hence, it seems the relationship between the levels of helping and ecological background may differ for different species.

The group size was also reported to be related to the territory size. Individuals may adjust territory size to ensure the resources necessary for survival and breeding (Dunk & Cooper, 1994; Gass, 1979). Two conflict patterns were detected among different studies. A positive correlation occurred between the number of group members and territory size of White‐banded tanagers (Duca & Marini, 2014). In the Cinnamon‐breasted rock bunting *Emberiza tahapisi*, however, the flock size was negatively related to territory size; but positively related to food abundance (Atuo & Manu, 2013). Territory density, refers as the average number of territories per unit area of suitable habitats, is the reciprocal of the mean territory size. The increasing of territory density would usually lead to the decreasing of territory size. While, there is a minimum size of territory to supply the needs of individuals (Hixon, 1980; Perrins & Birkhead, 1983). As expected, the group size was found to be increased with territory density in blue korhaan *Eupodotis caerulescens* (Moreira, 2006).

However, the group sizes, territory size or density, prey availability and rainfall were all transient parameters measured at a particular point in time in all of the above studies, what in fact are they fluctuate across seasons or years (Emlen, 1982; Ke, 2009; Komdeur, 1992; Rubenstein, 2016). Thus, the question arises whether there are more stable parameters to measure group size and these ecological factors. One case has been reported. A population of Seychelles warblers was maintained at approximately 300 birds and 120 groups in the isolated Cousin Island with an area of 29 ha, when it reached the upper limit of carrying capacity (Komdeur, 1992; Komdeur, Burke, Dughale, & Richardson, 2016), which was different from that of their populations in other islands (Komdeur et al., 2016). Thus, we can directly infer that the long‐term MGS and mean group density (MGD) or bird density stayed relatively stable under a certain level of carrying capacity (Komdeur, 1992; Komdeur et al., 2016). In populations of territorial species, individuals are usually limited in social groups within certain territories. The surplus of ecological resources in one territory cannot be utilized by group members in other territories, and vice versa. As a result, the MGD is better than bird density to be used as a fitness measure in territorial bird species. Hence, we can hypothesize that the long‐term MGS should be correlated to long‐term MGD.

MGD is equal to mean territory density, which is also the reciprocal of mean territory size. Numerous studies have demonstrated that long‐term mean territory size is inversely related to food abundance per unit area (reviewed by Smith & Shugart, 1987). When individual fitness is maximized in habitat of optimal quality, habitat quality may be the ultimate regulator of territory size (Smith & Shugart, 1987). Thus, factors like food, nesting sites, cover, and predation rates would simply be components of the overall habitat quality (Franzblau & Collins, 1980; Fretwell & Lucas, 1969; Orians, 1971). In this way, the food abundance per unit area is one important indicator of habitat quality. It also suggests that mean territory size, mean territory density, and MGD can be all used as indicators of habitat quality (Holmes, 1970; Myers, Conners, & Pitelka, 1979; Seastedt & MacLean, 1979; Simon, 1975; Stenger, 1958). Additionally, long‐term MGD may be a better indicator of habitat quality compared with food abundance per unit area, because it is an ecological result of synthesized influences from nest sites, cover, predation, climate, etc. and not only from food abundance. Hence, the correlation between long‐term MGS and MGD can also explain how MGS varies with habitat quality. As shown in Ethiopian wolves *Canis simensis*, a social group's composition is determined by its territory quality (Tallents, Randall, Williams, & Macdonald, 2012). However, the correlation between long‐term MGS and MGD received little concern. The question remains regarding how MGS will change with habitat quality (as indicated by MGD) and over what periods “long‐term” MGD needs to be calculated, in order to provide a reliable predictor of habitat quality.

The Masked laughingthrush *Garrulax perspicillatus* is a bird species once placed in Timaliidae and now excluded as Leiothrichidae (Cibois, Gelang, & Pasquet, 2010; Gelang et al., 2009), which is widely distributed throughout southeastern China (Zhao, 2001; Zheng, 2011). Although the breeding ecology of Masked laughingthrushes had been recorded (Liu, Jia, & Ning, 2002; Ma, 1989), their cooperatively breeding behaviors were recently confirmed (Ke, Long, Huang, Liao, & Hu, 2011). The birds live in social groups within year‐round territories. Their suitable habitats are broken into pieces, which are scattered among residential area, paddy fields, water bodies, and hilly areas (Ke et al., 2011).

In this study, based on six years’ field observation on a population of Masked laughingthrushes, we recorded their group sizes and territory distribution. We measured the areas of all fragmented plots of suitable habitats, and finally, we explored the correlation between MGS and MGD and aimed (1) to show how MGS varied with MGD and (2) to explore the lower limit of time duration that can be tested for a stable correlation between MGS and MGD.

## 2 MATERIALS AND METHODS

### 2.1 Study area and climate

**Figure 1:**
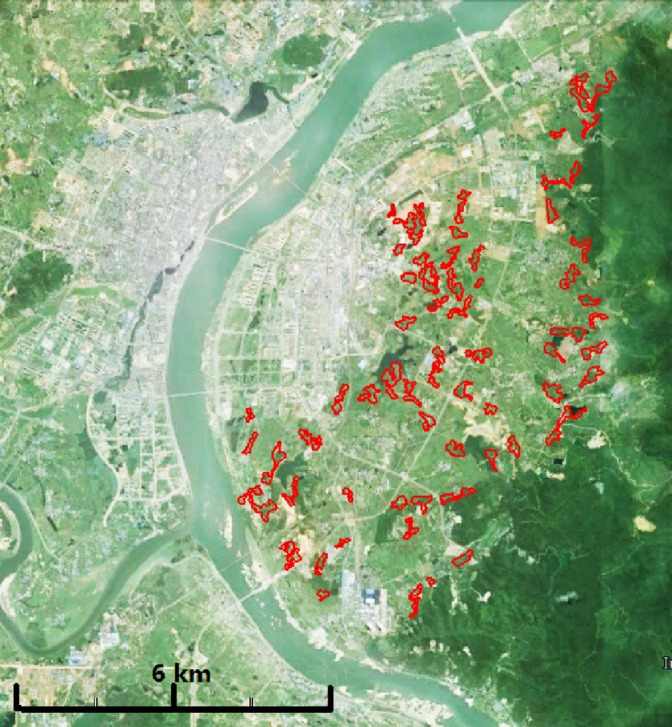
The distribution of suitable habitats (red circles) of the population distribution of Masked laughingthrushes in a geographical map

**Figure 2:**
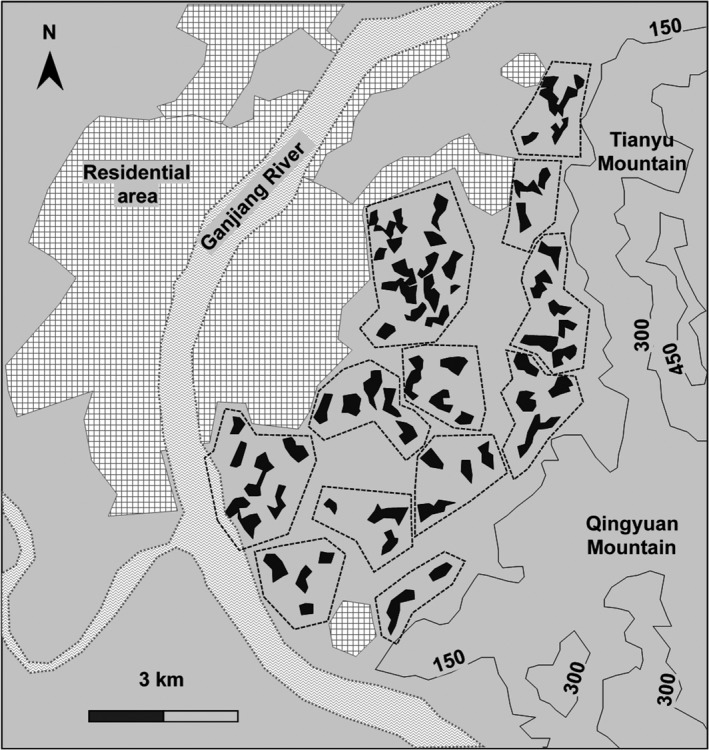
A sketch map of the population distribution of Masked laughingthrushes showing the fragmentation of suitable habitats and the relative isolation by surrounding environments. Black areas indicate the fragmented suitable habitats, which are interlaced with small villages, paddy fields and lower hills and surrounded by Tianyu‐Qingyuan Mountain, Ganjiang River and residential area of Ji'An city. Dashed boxes illustrate the division of the 12 subpopulations and subareas. Lines with numbers show the topographic contour line of 150 m, 300 m, and 450 m of the two mountains

Field work was carried out in the suburbs of Ji'An (27°06′N, 115°02′E), Jiangxi Province, China, from 2010 to 2016. The study area is bounded by Tianyu‐Qingyuan Mountain with an average altitude 300 m to the east and Ganjiang River in the other three directions (Figures 1 and 2). Residential areas were interlaced with hilly grounds, water bodies, and paddy fields with an average altitude of 75 m in the whole study area. The forest environments where Masked Laughingthrushes lived were broken into pieces, unevenly and discretely distributed in the whole study area (Ke et al., 2011). Climatic data were collected at a weather station (no. 57799) at the southwest of the study area. A subtropical monsoon climate is the typical climate, and the long‐term (1980–2016) average annual temperature was 19.0°C, with a minimum of 6.5°C in January and a maximum of 30.5°C in July. Annual total precipitation was 1504 mm, 80% of which fell between January and August.

### 2.2 Group size counting

Masked laughingthrushes initiate reproduction in February and end in August of each year. During each breeding season, we surveyed the whole study area territory by territory and observed all social groups by conducting song playback. The songs were collected from populations out of the study area. In this study, it is difficult to color‐band all the birds, especially the adults, and there was also no DNA analysis for member relationships. While some birds were marked during nestling stage, they were observed staying at home with their parents in the next year. This suggested that group members are families. These family groups showed strong adherence to their territories and actively defended them. When we played the songs of the birds, Masked laughingthrushes shyly responded in the early breeding season (March to May) but reacted strongly in the other stages. When there was only one group in the censusing area, we enticed them to a relatively open area for easy bird counting. In some situations, there could be more than one group appeared after the playback, these groups reacted not only to the songs we played but also strongly to the each other. They flew to their territory boundaries and defended it by active singing. If there was no actual invasion, they would gradually return to a normal state and retreat from the boundaries. Hence, it is not difficult for us to distinguish the territory boundaries of each family group and the individual affiliations.

Group size was the maximum number of birds in each group territory among all the visual observations in a same season (which could have included repeated observations of the same group of birds on different days). The group sizes based on more observations were considered to be more reliable (in single observations, some individuals could have been missed). Group sizes of the birds may fluctuate in different seasons, and different groups may initiate their reproduction asynchronously. The birds behaved in a strongly skulking manner due to nest protection in the early breeding season (February to May), which led to difficulty in counting group size for all the groups. In this study, we use the data of group sizes in the latter breeding season (June to August), in which season the birds respond more actively to playback, and the data of group sizes were more complete than those in the early breeding season. In this study, fledglings born in the current season were excluded in the group sizes, because they did not participate in territory defense or group formation at the start of the breeding season. These fledglings can be easily distinguished by the traits of lighter feather color and timid behavior in their early fledging stage.

### 2.3 Habitat suitability and measurement

According to characteristics of vegetation composition and spatial structure, the habitat in our study area can be roughly distinguished into three types of compartmental patches with relatively distinct boundaries: (1) dense living grasses or bushes on the ground with dense or sparse trees; (2) bared grounds with sparse trees; and (3) sufficient ground litter under sparse trees. Masked laughingthrushes were usually foraging and breeding in those habitats characterized by sparse trees and a small amount of litter. All these types of patches in which we observed the behaviors of foraging and breeding of the birds (bird presence) more than three times were considered suitable habitats for the birds. The territory was defined as the area used for living and breeding (Maher & Lott, 1995). Territories of different social groups were distinguished by territory defense behavior and the natural boundaries of the patches where there were no neighboring groups. Based on field observations of the natural boundary of habitat patches and territory defensive behaviors, we finally delineated the distribution map of the birds (Figures 1 and 2). We were then able to measure the areas of all the patches of suitable habitats. All the areas were measured using Google Earth Pro software.

### 2.4 Subarea/subpopulation division

For territorial bird species, spatially closed social groups may be close relatives due to limited dispersal. They breed independently in summer but may amalgamate during a harsh winter, while communications between those spatially distant social groups may be limited due to territorial defense or the long distance between territories (Ke, 2009). Intraspecific competition for resources, one of the main density‐dependent processes, will work mainly on a local scale in territorial species (Newton, 1992). Hence, for a territorial bird species living in strongly fragmented habitats, distant habitat patches can be seen as relatively independent land islands, in which the utilization of ecological resources is relatively exclusive. Similarly, distant groups or territories can be considered relatively independent. In contrast, neighboring habitat patches may be shared by neighboring groups across different seasons through group amalgamation or generational replacement. Although the members of the social groups may change across years or seasons, these neighboring habitat patches can be looked as a whole, which is shared by the continuum of family generations of these neighboring social groups. Correspondingly, the continuum can be looked as a subpopulation. Hence, the perspective can be changed as to how a subarea of the suitable habitat supports the social groups in the subpopulation across the years, regardless of who the group members are and where they are from.

Masked laughingthrushes live year‐round in territories within social groups, and their suitable habitats are partitioned by large areas of paddy field, water bodies, and higher mountains (Ke et al., 2011; Figures 1 and 2). Among these fragmented patches of suitable habitats, one family may occupy several patches, and one patch may also sometimes include several territories. In total, there were approximately 50 family groups scattered among these patches each year with an average nearest distance of 600 m (ranging from 230 m to 1,200 m) between territories (Ke et al., 2011). Their territories may vary across the years because of new territory foundation, old territory shifting, territory budding, disappearing, and amalgamation. We recorded the geographic coordinates of the central points of all the territories over the six‐year period. Based on the location information, we clustered the contiguous habitat patches into subareas using the centroid linkage and clustered corresponding social groups across years into subpopulations. The total area of all the patches in a subarea is considered to be the area of suitable habitats for the subpopulation in the subarea.

### 2.5 MGS and MGD averaged for different time durations

Mean group size and mean group density for the subpopulations in certain subareas were calculated by the data combined for each year or two to five consecutive years. The MGDs were calculated as the mean number of social groups divided by the total area of suitable habitats in each subarea. We marked the MGS as *Gx* (G0, G2… and G23, G34…, etc. represented the MGS data of the year 2010, 2012… and 2012‐2013, 2013‐2014…, etc.), and MGD as *Dx* (similarly, D0, D2… D23, D34 …D023456 to represent the MGD for corresponding time durations). Because the data in the year 2011 were not included, the data of 2010 were only used to calculate G0, D0 for 1 year, but G023456 and D023456 were used for all 6 years in this study.

### 2.6 Data analysis

Using MGD as independent variables and MGS as the dependent variable, the response curves of MGS to MGD are fitted by a quadratic function and modeled by quadratic regression. First, we explore the correlations between *Gx* and D023456 to show how MGS varied with long‐term MGD. Second, we explore the correlation between *Gx* and *Dx* with same time durations (*x*) and try to find a lower limit of years for the stable correlation that can be tested. Finally, we made a *p*‐value matrix for the correlations between *Gx* and *Dx* (related combination by changing the time duration (*x*) for both parameters) to show how MGS varied with MGD. When the correlations between MGS and MGD for all combinations of a certain time duration (from one to six years) were significant, the correlation was considered to be stable, and the time duration was considered that required for a stable correlation that can be tested. The smallest of these time durations is considered to be the lower limit of the time duration for the “long term.”

Differences in group sizes among subpopulations or across years were examined using one‐way ANOVAs. All statistics were performed using SPSS software 20.0 (IBM, Armonk, NY, USA). *R* ^2^ values of all correlations are provided. Probabilities are two tailed, and the significance level is set at α = 0.05. All values given were mean ± 1*SD*.

## 3 RESULTS

### 3.1 Suitable habitats and Subarea division

The total area of suitable habitats was 247 ha, which is fragmented and scattered in the whole study area (120 km^2^, Figures 1 and 2). During the 6 years, we recorded a total of 110 geographic coordinates of different locations of territories due to the fluctuation of territory ranges. Using the centroid linkage, we clustered all these locations into 12 subgroups. The corresponding area of suitable habitats around these locations was divided into 12 subareas (Figure 2). The mean area of the suitable habitats for the 12 subareas was 20.59 ± 10.51 ha. Correspondingly, the whole study population was broken into 12 subpopulations.

### 3.2 Group sizes among subpopulations

During the 6 years (2010, 2012–2016), we observed a total of 309 social groups in the summer, among which were 266 families with data for group sizes. The average group size of the whole population was 3.88 ± 1.19 individuals. The proportion of families with more than two members was 89.1% (237/266). The group sizes were significantly different across years (*F*_5, 260_ = 3.862, *p *=* *.002) and among the 12 subpopulations (*F*_11, 254_ = 1.863, *p *=* *.045).

### 3.3 Area effect of suitable habitat on the number of social groups

**Figure 3:**
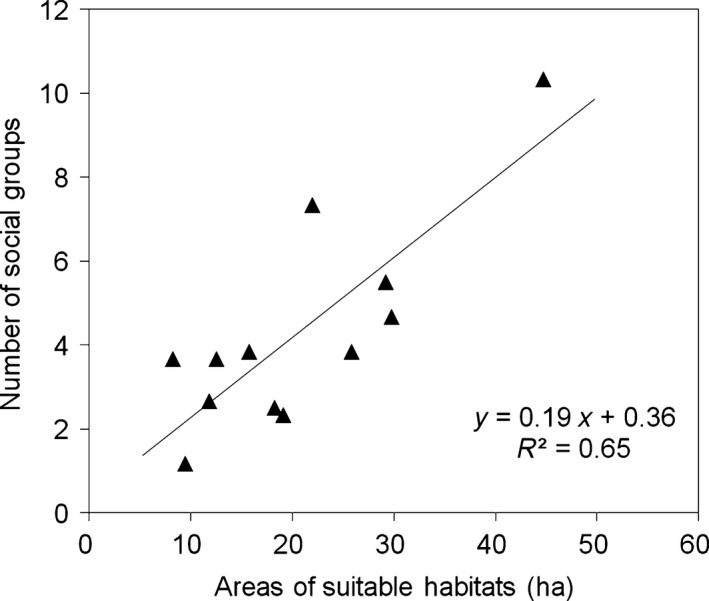
Long‐term mean group numbers plotted against the total area of suitable habitats for the 12 subpopulations in the 12 subareas

At the subpopulation level, the annual mean number of social groups was 4.29 ± 2.48 groups for the 6‐year period. For all subpopulations in the 12 subareas, the total area of available suitable habitats was positively correlated to the long‐term annual mean numbers of social groups (*R* ^2^ = 0.652, *F* = 18.72, *p *=* *.001; Figure 3).

### 3.4 Correlation between MGS and MGD

**Figure 4:**
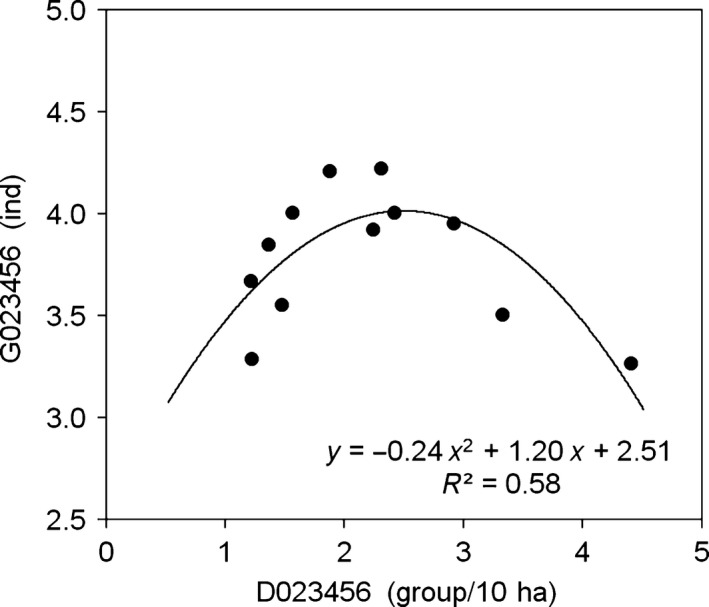
The long‐term mean group size (MGS) in Masked laughingthrushes plotted as a quadratic function of long‐term mean group density (MGD). Both long‐term MGS and MGD were calculated by pooled data of all 6 years (G023456 vs. D023456)

First, we explored the quadratic correlation between MGS and MGD (MGS = *b*_0_ × MGD^2^ + *b*_1_ × MGD + *b*_2_) using D023456 (MGD for all the 6 years) as the independent variable and MGS for different time durations (G0, G2, etc.) as the dependent variables. The results showed that D023456 is significantly related to G2, G3, G02, G23, G45, G234, G345, G456, G2345, G3456, G23456, and G023456 but not to G0, G4, G5, G6, G34, and G56 (Table 1). Obviously, when the group sizes were averaged for three or more years, MGS is significantly and stably related to long‐term MGD (D023456) in a reverse parabolic association. The correlation between G023456 and D023456 is MGS = −0.240 × MGD^2^ + 1.201 × MGD + 2.515 (*R* ^2^ = 0.584, *F* = 6.313, *p *=* *.019; Table 1, Figure 4).

**Table 1:** Statistical test results of the quadratic correlation between mean group size (MGS) and mean group density (MGD) according to different time durations. Legends: see Table 2

Time duration	*Gx* a	*Gx* vs. D023456			*Gx* vs. *Dx*		
		*R* ^2^	*F*_2,9_	*p*	*R* ^2^	*F*_2,9_	*p*
One year	G0	0.157	0.839	.463	0.085	0.418	.671
	G2	0.767	14.774	.001	0.467	3.939	.059
	G3	0.509	4.668	.041	0.291	1.843	.213
	G4	0.073	0.353	.712	0.116	0.591	.574
	G5	0.337	2.289	.157	0.09	0.445	.654
	G6	0.307	1.993	.192	0.608	6.981	.015
Two consecutive years	G23	0.844	24.371	.000	0.519	5.06	.034
	G34	0.256	1.550	.264	0.327	2.183	.169
	G45	0.489	4.309	.049	0.262	1.595	.255
	G56	0.424	3.307	.084	0.327	2.187	.168
Three consecutive years	G234	0.706	10.794	.004	0.679	9.500	.006
	G345	0.593	6.547	.018	0.438	3.513	.075
	G456	0.550	5.506	.027	0.396	2.955	.103
Four consecutive years	G2345	0.649	8.315	.009	0.590	6.487	.018
	G3456	0.558	5.681	.025	0.529	5,064	.034
Five consecutive years	G23456	0.589	6.452	.018	0.607	6.964	.015
Six years	G023456	0.584	6.313	.019	0.584	6.313	.019

a Code legends provided in Table 2.

Assuming that the study period is only one or two to five consecutive years among the years from 2010 to 2016, we explored the quadratic correlation between *Gx* and *Dx* (with the same time durations). The results showed that the correlations were significant between G6 vs. D6, G23 vs. D23, G234 vs. D234, G2345 vs. D2345, G3456 vs. D2345, and G23456 vs. D23456 (Table 1). Therefore, when the group densities were both averaged for more than 3 years, the significant correlations between MGS and MGD remained stable.

Based on the above findings, we made a matrix of the *p* values of the correlations between MGS and MGD, both of which were cross‐related with different time periods (Table 2). The matrix showed us that when MGS was averaged for one or two years and MGD for one to three years, the correlations between MGS and MGD were tested to be occasionally significant for those time durations. When the MGS was averaged for more than 2 years and MGD for more than 3 years, the *p* values were all smaller than 0.05 (except G456 vs. D3456, *p *=* *.051). Such results supported the hypothesis that if we calculated the MGS for more than 2 years and MGD for more than 3 years based on the division of 12 subareas, then significant constant quadratic correlations between MGS and MGD can be detected.

**Table 2:** The *p* value matrix of the quadratic correlations between mean group size (MGS) and mean group density (MGD). It was cross‐correlated between *Gx* and *Dx* with different time durations (1–6 years). Dark grids show significant correlation at *p* = .05. *G* indicates mean group size, *D* indicates MGD, and the numbers from 0 to 6 following *G* or *D* represent the corresponding time durations for each year or consecutive years from 2010, 2012 to 2016; see also in text

MGS vs. MGD	D0	D2	D3	D4	D5	D6	D23	D34	D45	D56	D234	D345	D456	D2345	D3456	D23456	D023456
G0	0.671	0.397	0.079	0.184	0.642	0.647	0.248	0.223	0.276	0.506	0.324	0.296	0.345	0.391	0.376	0.464	0.463
G2	0.274	0.059	0.169	0.001	0.022	0.002	0.077	0.000	0.002	0.002	0.001	0.001	0.002	0.001	0.001	0.001	0.001
G3	0.980	0.034	0.213	0.064	0.260	0.045	0.108	0.050	0.084	0.045	0.038	0.064	0.051	0.048	0.049	0.042	0.041
G4	0.540	0.997	0.183	0.574	0.528	0.845	0.803	0.285	0.594	0.802	0.619	0.402	0.715	0.624	0.531	0.684	0.712
G5	0.031	0.060	0.180	0.687	0.654	0.221	0.136	0.489	0.675	0.386	0.197	0.503	0.484	0.229	0.360	0.191	0.157
G6	0.486	0.423	0.632	0.167	0.533	0.015	0.320	0.253	0.338	0.175	0.137	0.366	0.154	0.212	0.190	0.132	0.192
G23	0.690	0.011	0.096	0.001	0.025	0.000	0.034	0.000	0.002	0.001	0.000	0.001	0.001	0.000	0.000	0.000	0.000
G34	0.431	0.649	0.563	0.243	0.188	0.235	0.810	0.169	0.239	0.265	0.329	0.207	0.253	0.306	0.215	0.284	0.264
G45	0.097	0.102	0.233	0.244	0.275	0.126	0.077	0.083	0.255	0.182	0.033	0.115	0.188	0.043	0.088	0.044	0.049
G56	0.224	0.061	0.605	0.408	0.461	0.068	0.156	0.349	0.418	0.168	0.101	0.346	0.229	0.117	0.199	0.084	0.084
G234	0.230	0.177	0.388	0.003	0.010	0.004	0.255	0.001	0.004	0.005	0.006	0.003	0.004	0.005	0.003	0.004	0.004
G345	0.376	0.309	0.674	0.068	0.253	0.006	0.315	0.033	0.126	0.048	0.026	0.075	0.049	0.045	0.029	0.021	0.018
G456	0.118	0.098	0.295	0.145	0.254	0.040	0.082	0.059	0.186	0.103	0.019	0.091	0.103	0.028	0.051	0.022	0.027
G2345	0.403	0.327	0.726	0.017	0.146	0.003	0.331	0.009	0.043	0.021	0.009	0.025	0.018	0.018	0.011	0.009	0.009
G3456	0.273	0.369	0.675	0.069	0.297	0.006	0.331	0.037	0.143	0.060	0.028	0.086	0.056	0.048	0.034	0.024	0.025
G23456	0.275	0.421	0.772	0.028	0.215	0.005	0.370	0.015	0.073	0.038	0.014	0.044	0.031	0.028	0.019	0.015	0.018
G023456	0.390	0.415	0.339	0.013	0.242	0.014	0.200	0.005	0.050	0.045	0.007	0.025	0.026	0.019	0.016	0.015	0.019

## 4 DISCUSSION

### 4.1 The volatility and stability of population dynamics

As shown with the Seychelles warblers, the populations fluctuated across years but remained stable at an average level of approximately 120 groups and 300 individuals, when it reached the upper limit of carrying capacity (Komdeur, 1992), similar patterns were found in other populations (Komdeur et al., 2016). These populations showed us the effects of both volatility year by year and stability averaged by certain time durations. Previous studies tried to explore how the group size varied with ecological gradients, while both parameters for the populations were measured for just one certain season or year (Emlen, 1982; Ke, 2009; Komdeur, 1992). The inconsistent results in the above studies might be attributed to the effects of volatility across years (Reyer, 1980). In this study, the effects of both volatility and stability are explored. When the MGS is averaged for <3 years and the MGD for <4 years in populations of Masked laughingthrushes, the quadratic association between MGS and MGD is unstable, although it is occasionally significant (Table 2). The correlations were shown to be positive, negative, or quadratic as short‐term effects in different situations according to different time durations. Correspondingly, they are similar to those studies as simply positive (Komdeur, 1992; Rubenstein, 2016) or negative correlation (Emlen, 1982; Ke, 2009). In contrast, when MGS is averaged for more than 2 years and MGD for more than 3 years, a reverse quadratic correlation between long‐term MGS and MGD becomes more constant (Table 2, Figure 4).

The external ecological factors may play important roles in the social structure and group dynamics, but their effect can be delayed. The current population state may be determined by ecological factors in the past; as shown in Tibetan ground tits, the current extreme drought led to intensified cooperation in the next year (Ke, 2009). The transient group density is not just a result of current ecological conditions but a comprehensive result from ecological stresses experienced in at least several seasons or years (Batten & Marchant, 1977; Marra & Holmes, 2001). Due to the effects of the delayed ecological influences and the asynchrony of such influences among different populations, the volatility is understandable. However, when all the parameters are averaged for a sufficient elapsed time, it will show us the long‐term effects as stability. Such stability is sourced from the correlation between the population dynamic and long‐term habitat quality or the carrying capacity per unit area. A stable quadratic correlation between long‐term MGS and long‐term MGD in Masked laughingthrushes demonstrates this stability.

### 4.2 The reason for a quadratic correlation

In Masked laughingthrushes, when MGS is averaged for more than 2 years and MGD for more than 3 years, long‐term MGS is correlated with long‐term MGD in a reverse quadratic shape. This suggests that the long‐term MGS varied with the gradient of long‐term habitat quality as the smallest in the best or worst habitats but highest in the moderate state. The question then arises as to the reason for the quadratic relationship.

Given that there are three prerequisites: (1) In the wild population, individuals will pursue independent reproduction to maximize the reproductive benefits in their lives to the greatest degree possible (Both & Visser, 2000); (2) Wild populations have a higher survival rate under better habitat quality and a lower survival rate in worse habitats; (3) Mortality is different between sexes (Newton, 1998; Sinclair, 1989). Females usually have higher mortality than males under extreme harshness (Ke, 2009), but it may be the inverse in some cases. Then, the quadratic correlation can be explained by deduction from the conditions given above for three different situations. (1) When a population lives under conditions of extreme lower habitat quality, (a) the competition among adults would be intensified; (b) higher mortality for both males and females in the whole population; while, (c) females have the lower survival than males. Under such a state, the population has less number of groups with the smallest MGS. (2) When a population lives under the best habitat quality, (a) the competition among individuals is alleviated; (b) the mortality is lower for both males and females; and (c) the sex ratio is close to 0.5. Under such a situation, all individuals try their best to breed independently, and the population will have larger number of groups with the smallest MGS. (3) When a population lives under moderate habitat qualities, (a) competitions among individuals are moderate; (b) mortality is higher in females than that in males; and (c) the sex ratio of the population is highly skewed toward males. In such a situation, the population has a larger MGS. In short, more pairs would breed independently in the better condition, while there would be limited number of pairs can breed independently in the worst conditions; in moderate conditions, skewed sex ration would lead to larger group sizes.

### 4.3 Long‐term MGD: a better index for habitat quality

It is often difficult to find a good measure of habitat quality for most species or populations. Some researchers have used time budgets to reveal favored habitat types (Brown & Balda, 1977), vegetation structure (Brown & Brown, 1981), or a measure of the available food resources (Macroberts & Macroberts, 1976; Trail, 1980) as potential indices of territory quality in different species. None of the above indices can be generally used to measure the habitat quality for all species. However, we can expect the long‐term MGD to be a better index for habitat quality in populations of territorial species, due to following reasons.

Long‐term MGD is the synthesized results of all ecological factors across years. Many studies suggested that long‐term mean territory size is inversely related to food abundance per unit area (Holmes, 1970; Myers et al., 1979; Seastedt & MacLean, 1979; Simon, 1975; Smith & Shugart, 1987; Stenger, 1958), and thus, it is inversely related to long‐term MGD. Because the food abundance per unit area is one important indicator of habitat quality (Emlen, 1982), both long‐term mean territory size and long‐term MGD can also be used as indicators of habitat quality. And in fact, the long‐term MGD is a synthesized result of food distribution, forage investment, nest site selection, habitat saturation, and population dynamics in the wild populations of territorial species, which are the components of overall habitat quality (Franzblau & Collins, 1980; Fretwell & Lucas, 1969; Orians, 1971). Carrying capacity of certain area of habitats is affected by the abundance and distribution of resources and by how individuals compete for these limiting resources (Ayllon, Almodovar, Nicola, Parra, & Elvira, 2012; Rees, 1992). The stable quadratic correlation between long‐term MGS and MGD in this study also supports the hypothesis that long‐term MGD can be used as an indicator of the long‐term habitat quality, and the period of the “long term” is a time duration of three or four years in Masked laughingthrushes. In addition, MGD is an indicator that is more easily to be recorded and estimated compared with other detailed ecological factors.

The long‐term MGD has reflected that the natural resources in certain area of suitable habitats are utilized together by all social groups and their generations. For one certain social group, all the members exclusively utilize the resources in their territory in certain seasons. For different social groups, neighboring ones may breed independently in summer but amalgamate in winter; new groups may bud from territories of their parents, and one group may expand their territory to neighboring territories after the original “owners” disappeared (Ke, 2009). While those distant social groups have very limited influences on natural resources in the other's habitats. Hence, all the natural resources across years in the certain area of habitats were utilized together by those social groups and their generations living in the habitat. The long‐term MGD reflects the upper limit of carrying capacity at group level.

### 4.4 A theoretical consideration

Although cooperative breeding has engendered considerable interest among behavioral ecologists since Skutch (1935), we are far from understanding the evolution of cooperative breeding (Hatchwell, 2009; Koenig & Dickinson, 2004, 2016), and conflicting conclusions are still presented in different studies (Ke & Huang, 2010; Koenig, Dickinson, & Emlen, 2016; Pruett‐Jones, 2004). The quadratic association between long‐term MGS and MGD brings us new insights into the evolution of cooperative breeding.

Firstly, it is long‐term habitat quality (MGD) determined long‐term MGS in certain habitats, as suggested in the population of masked laughingthrushes. In other words, the correlation between long‐term MGS and MGD sets no prerequisites regarding who the group members are and of which relationship they are. The majority of helpers were shown to be the close relatives of the breeders they helped in cooperative breeding systems (Emlen, 1997; Dickinson & Hatchwell, 2004; Ke, 2009), or some of them were nonkin (Cockburn, 1998; ,Clutton‐Brock, 2002; Nomano et al., 2015) or even kidnapped members from nonkin (Ridley, 2016). If there was no prerequisite for helper identity and status, the formation of the social groups would be ecologically based, not causally determined by individuals themselves and their relationships.

Secondly, for certain population living in certain habitat, long‐term MGS is adapted to its corresponding long‐term habitat quality (MGD), although the group sizes fluctuated across years. If so, helper effects can be deduced for different situations. When the current group sizes are smaller than long‐term MGS, the helping can be tested to be beneficial to the social groups in both survival and reproduction. When the current group sizes are larger than long‐term MGS, a reverse tendency can be expected (Brouwer, Richardson, Eikenaar, & Komdeur, 2006). However, long‐term effects may have the opposite tendency because increasing survival and reproduction will increase competition in the future, when the whole population reaches its upper limit of carrying capacity (Brouwer et al., 2009). As shown in previous studies, the helper effect was positive (Brooker & Rowley, 1995; Davies & Hatchwell, 1992; Doerr & Doerr, 2007; Woxvold & Magrath, 2005) and negative (Heinsohn & Cockburn, 1994; Koenig, 1990), co‐existence of both positive and negative (Ke, 2009) or with no significant influences (Eguchi, Yamagishi, Asai, Nagata, & Teruaki, 2002; Legge, 2000; Leonard, Horn, & Eden, 1989; Magrath & Yezerinac, 1997). Such diversity in helper effects is easy to be understood.

## AUTHOR CONTRIBUTIONS

D.‐H. Ke designed the study. D.‐H. Ke, Y.‐H. Deng, W.‐B. Guo, and Z.‐H. Huang performed field work. D.‐H. Ke performed data analysis and writing by.

## CONFLICT OF INTEREST

None declared.
